# Secure Messages, Video Visits, and Burnout Among Primary Care Providers in the Veterans Health Administration: National Survey Study

**DOI:** 10.2196/68858

**Published:** 2025-09-05

**Authors:** Eric A Apaydin, Claudia Der-Martirosian, Caroline Yoo, Danielle E Rose, Nicholas J Jackson, Susan E Stockdale, Lucinda B Leung

**Affiliations:** 1 Department of Medicine, David Geffen School of Medicine, University of California, 11301 Wilshire Blvd, Los Angeles, CA, 90073, United States, 1 3104783711 ext. 44860; 2 Center for the Study of Healthcare Innovation, Implementation and Policy, VA Greater Los Angeles Healthcare System, Los Angeles, CA, United States; 3 RAND Corporation, Santa Monica, CA, United States; 4 Veterans Emergency Management Evaluation Center, Veterans Health Administration, North Hills, CA, United States; 5 Department of Health Policy and Management, Fielding School of Public Health, University of California, Los Angeles, CA, United States; 6 Department of Psychiatry and Biobehavioral Sciences, David Geffen School of Medicine, University of California, Los Angeles, CA, United States

**Keywords:** primary care, primary care provider, telehealth, Veterans Health Administration, VHA, emotional exhaustion, depersonalization

## Abstract

**Background:**

Telehealth use, including video visits and secure messages, expanded significantly in Veterans Health Administration (VHA) primary care during the COVID-19 pandemic. However, primary care provider (PCP) burnout also increased during this period. Each modality may have affected primary care workloads differently (either by substituting for or complementing in-person visits) and thereby had varying effects on PCP burnout.

**Objective:**

This study aims to examine the associations between PCP burnout and the volumes of video visits and secure messages within the health care systems in which the PCPs practiced.

**Methods:**

This study examined the associations between telehealth modalities (ie, video visits and secure messages) and burnout as reported by 17,034 PCPs in 138 health care systems in VHA from 2020 to 2023. Individual-level data were obtained from annual cross-sectional surveys, and health care system–level data were drawn from administrative data sources. We created logistic regression models using generalized estimating equations to analyze the relationships between individual-level PCP burnout and average volumes of health care system–level video visits and secure messages per 1000 patients, controlling for age, sex, race or ethnicity, and VHA tenure as well as health care system complexity and year. We then predicted the marginal means of PCP burnout by video visit or secure message volume, based on the model results.

**Results:**

From 2020 to 2023, average PCP burnout, across repeated, annual cross-sections, increased from 42.1% to 52.7% (survey response rates of 68%‐74%). Most survey respondents were aged 50 years and above (9607/17,034, 56.40%), female (10,189/17,034, 59.82%), non-White (9460/17,034, 55.54%), and with less than 10 years of tenure in the VHA (10,990/17,034, 64.52%). Over these 4 years, median annual video visits per 1000 patients in health care systems increased from 15.9 (IQR 8.4-25.5) to 227.6 (IQR 127.1-320.7), and median annual secure messages per 1000 patients increased from 23.4 (IQR 9.4-65.5) to 35.3 (IQR 11.0-87.0). In our fully adjusted models, video visit volumes in a health care system were not related to burnout, but secure message volumes were related to burnout. Burnout was significantly higher among PCPs in health care systems receiving additional secure messages per 1000 patients (odds ratio 1.001, 95% CI 1.000-1.002). On average, PCP burnout increased by 1% point for each additional increase of 43.7 (95% CI 14.0-73.4) secure messages in a health care system.

**Conclusions:**

Video visit volumes in a health care system were not associated with PCP burnout, but secure message volumes were associated with PCP burnout. As video visits and secure messages continue to grow, solutions to better manage message volume (eg, automation and provider-led quality improvement) are needed to mitigate the concurrent rise in PCP burnout.

## Introduction

### Background

Burnout among primary care providers (PCPs) in the United States has long been persistently high [1-6] and has recently been recognized as a national crisis [7,8]. Drivers of burnout are multifactorial, including excessive workloads or work hours, poor leadership quality, weak team structure, and high clerical or administrative burden, including the complexity and time-intensiveness of electronic health care record systems [2,4]. Telehealth in primary care has expanded dramatically during [9] and after [10] the COVID-19 pandemic, but it is unknown if, like electronic health care records, those visits affected PCP burnout.

Telehealth in primary care in the Veterans Health Administration (VHA), including synchronous video visits and asynchronous secure patient messages, has grown significantly since the pandemic. Video visits increased by 2 to 6 times during the early pandemic, and secure message use increased by about 50% [11]. Even as in-person care stabilized in 2023, patients still attended about 60,000 video primary care visits per month in the VHA [12]. Patients are generally satisfied with video visits [13,14] and secure messages [15] in primary care, but PCPs’ perceptions of these technologies are more mixed [16-18]. Video visits are direct, synchronous patient care, so they may act as substitutes to in-person visits. On the other hand, secure messaging is asynchronous, and it may complement or add to video, phone, or in-person visits.

This complementary nature of messaging can lead to an increase in workload with the rapid expansion of telehealth services [19], adding to the already long list of administrative tasks (eg, placing orders, writing prescriptions, and reviewing clinical notes) undertaken by PCPs [20]. Virtual video visits may be less likely to increase the workload, as they substitute for in-person visits. As previously shown, workload and administrative burden are associated with PCP burnout [2,4]. The unstructured and potentially unlimited nature of secure messages may also harm work control and therefore increase burnout. According to the job demands-resources model of burnout, job demands (eg, workloads) and resources (eg, work control) compete to harm and motivate workers, ultimately resulting in burnout if the demands are too high [21,22]. In this study, we hypothesize that increases in secure messaging, but not video visits, may also be associated with an increase in PCP burnout.

Telehealth use in VHA varies by health care system [11], due to health care system–level policies that promote or discourage the use of telehealth, a variation in the offering of telehealth appointments to patients by scheduling clerks and PCPs, and differences in telehealth demand created by differences in in-person appointment availability. Therefore, we chose to model telehealth use at the health care system level to account for these differences in policies, personnel, and preferences.

### Objectives

Video visit and secure messages volumes increased dramatically during the COVID-19 pandemic, but their effects on primary care workload, and therefore burnout, may not be equivalent. Here, we present the first and largest national analysis of telehealth use and burnout in primary care, using a survey of over 17,000 PCP responses, to understand how clinician burnout varies by telehealth modality. Study objectives were (1) to examine the changes in video visits and secure messages handled by PCPs during and after the COVID-19 pandemic and (2) to analyze the relationships between PCP burnout and the average volume of video visits and secure messages in the health care system in which they practiced.

## Methods

### Study Design

We analyzed repeated cross-sectional data from 17,034 survey responses from PCPs in 138 health care systems (ie, hospitals and related outpatient clinics) in the VHA from fiscal year (FY) 2020‐2023 (FYs run from October of the previous year to September of the current year). The data contained individual-level survey responses (level 1) and health care system–level telehealth volume (level 2). Only complete cases were analyzed, and any with missing data were dropped.

### Data Sources and Sample Description

Our data sources included the Corporate Data Warehouse (CDW) [23] and the VHA All Employee Survey (AES) [24]. The CDW is a national VHA database that contains patient, clinical, enrollment, benefits, administrative, and financial data. The AES is an annual, VHA-wide survey of employee attitudes, usually administered in June (the FY 2020 administration was delayed until September). Response rates across the VHA were 70% in FY 2020, 68% in FY 2021, 70% in FY 2022, and 74% in FY 2023. Response volumes varied by year, and we included data from 3921 PCPs in FY 2020, 4296 PCPs in FY 2021, 4237 PCPs in FY 2022, and 4490 PCPs in FY 2023. The survey responses were anonymized and cross-sectional and may have been generated by different or the same respondents in each year. Our sample included physicians (MDs and DOs), physician assistants, and nurse practitioners who identified themselves as part of a primary care team (in the VHA, a Patient Aligned Care Team [PACT]).

### Outcome: Individual PCP Burnout (Level 1)

The main outcome for this study was PCP burnout, as evaluated by 2 single-item measures for emotional exhaustion (“I feel burned out from my work”) and depersonalization (“I worry that this job is hardening me emotionally”) in the AES. These measures, adapted from the Maslach Burnout Inventory, were assessed using symptom frequency on a 7-point scale (never, a few times a year or less, once a month or less, a few times a month, once a week, a few times a week, or every day). Burnout was operationalized as a binary outcome using the previously validated [25,26] and standard [1] method of defining burnout as reporting symptoms for either measure once a week or more (1=once a week, a few times a week, or every day; 0=never, a few times a year or less, once a month or less, or a few times a month).

### Exposures: Video Visit and Secure Message Volumes Within Health Care Systems in Which PCPs Practiced (Level 2)

Our main exposures were the average numbers of video visits and secure messages per 1000 primary care patients per health care system and year. We selected 1000 primary care patients as the unit of measure, representing an average panel size for one full-time PCP in the VHA. Telehealth volumes of video visits and secure patient messages were drawn from CDW data and aggregated to the VHA health care system in which an individual PCP respondent practiced. PCP responses were anonymized and could not be linked with individual PCPs and their telehealth practices; therefore, we first aggregated total visit counts at the PCP level and then further aggregated them to the health care system level. If PCPs visited multiple health care systems, we assigned them to the one health care system in which they most frequently practiced each year. As a result, video visit and secure message volume exposures represented aggregated annual averages of telehealth volumes in health care systems in which individual PCPs practiced, which may or may not reflect actual PCP telehealth use. However, as noted earlier, telehealth use in the VHA is promoted or discouraged at the health care system level, and so we found it appropriate to model telehealth use as this level. The use of video modality was identified using primary care clinic codes 648 and 679 and a current procedural terminology modifier for synchronous telemedicine service. The use of secure messages was identified using primary care clinic codes 189, 646, 647, 694, 695, 696, 698, 718, and 719 (Table S1 in Multimedia Appendix 1). To calculate annual visit or message counts per capita, we divided the number of video visits or secure messages by 1000 primary care patients per health care system and year. Both measures were continuous. Video visits were then modeled by quartiles: first quartile (0‐38.2 visits); second quartile (38.2‐140.1 visits); third quartile (140.1‐270.0 visits); and fourth quartile (>270.0 visits).

### Controls

#### Individual (Level 1)

This study used AES data to control for PCP characteristics that have been previously associated with self-reported burnout; specifically, age (30-39, 40-49, 50‐59, and ≥60 years); sex (male, female, and other or unknown); race or ethnicity (non-Hispanic White, non-Hispanic Black, non-Hispanic Asian, non-Hispanic other, non-Hispanic unknown, and Hispanic; race was significantly related to burnout in previous analyses of VHA PCPs [27,28]); and VHA tenure (less than or equal to 2 years, between 2 and 10 years, between 10 and 20 years, and ≥20 years) were included at the individual level.

#### Health Care System (Level 2)

Because health care system complexity may predict telehealth use, we controlled for this using a 3-category variable, the Veterans Health Administration Facility Complexity Model [29]. Group 1 health care systems (high complexity) typically have medium to high patient volumes, medium- to high-risk patients, more complex clinical programs, and medium to large teaching and research programs. Group 2 health care systems (medium complexity) typically have medium volume, low-risk patients, few complex clinical programs, and small or no teaching and research programs. Group 3 health care systems (low complexity) are similar to group 2 systems but with low patient volume and few or no complex clinical programs [30]. We also included the survey administration year (FY 2020, FY 2021, FY 2022, and FY 2023) as a fixed-effect control.

### Statistical Analysis

Logistic regression models using a generalized estimating equations approach were used to examine the associations between PCP burnout and video visits and secure message volumes at their respective health care systems, controlling for provider age, sex, race or ethnicity, VHA tenure, health care system complexity, and year. Provider characteristics were modeled at the individual level, while video visit and secure message volumes and health care system complexity were modeled at the health care system level. Predictive marginal mean proportions of burnout for a range of video visit and secure message volumes were also calculated using model results. The pairwise comparisons of these marginal estimates of burnout were made by the 0th, 75th, and 90th percentiles of video visit and secure message volumes.

In sensitivity analyses, we added health care system–level team staffing ratios and PCP staffing ratios as covariates. Teams were considered fully staffed if they had at least 3 staff (nurses, nursing assistants, and medical support assistants) per PCP, and health care systems were coded as *better staffed* (50%‐100% full staffing) or *very understaffed* (<50% full staffing). Health care systems were considered to be fully staffed with PCPs if the expected-to-observed panel size was ≥1.2, accounting for provider type (MD vs nurse practitioner or physician assistant) and provider time off, as previously described by O’Shea et al [31]. Health care systems that were not fully staffed had ratios less than 1.2.

Additional subgroup analyses were also conducted by sex for each model. *Z* tests for coefficient comparisons were used to compare odds ratios (ORs) between the male and female models.

For all models, we determined significance by using a 2-tailed α of .05 and analyzed data in Stata (version 18.0; StataCorp).

### Ethical Considerations

This project was part of an ongoing VHA quality improvement (nonresearch) effort and exempt from institutional review board review under 45 CFR 46.104(d)(5). The work was carried out as a quality improvement evaluation under the terms of a signed attestation of nonresearch from the VHA Office of Primary Care. This VHA documentation ensures that work not carried out under a human subjects protocol is part of institutionally sanctioned quality improvement activities. Informed consent was not applicable to this analysis as it was a secondary analysis of survey data and designated as nonresearch. All Employee Survey responses were anonymous and could not be linked to identifying information outside of the survey. Survey responses were voluntary and respondents were not compensated for their participation.

## Results

### Overview

**Figure 1.**
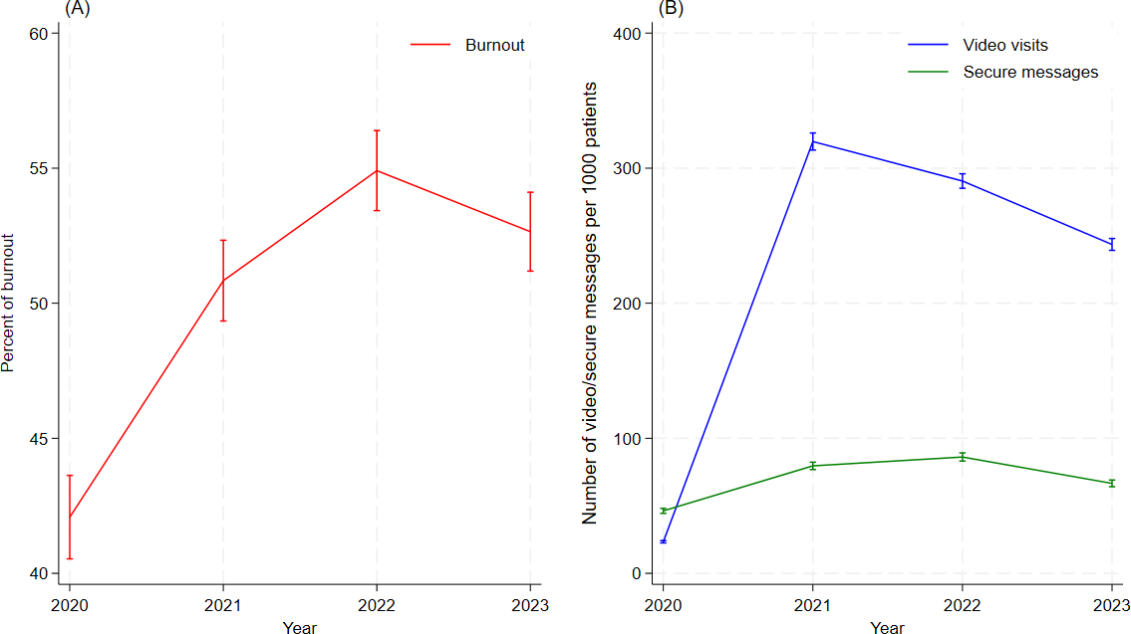
Burnout, video visits, and secure messages among Veterans Health Administration primary care providers by fiscal year. The figure depicts annual averages with 95% CIs at the health care system level. (A) Unadjusted trend of burnout by year. (B) Unadjusted trend of video visits and secure messages by year.

Nearly 50% of PCPs were burned out during our period of analysis, and that burnout increased from 42.1% in FY 2020 to 52.7% in FY 2023 across repeated, annual cross-sections (Figure 1). Across all years, burnout was generally higher among middle-aged (40‐59 years), White, female, and early career PCPs, as well as among those who worked in less complex health care systems (Table 1). Video visits per PCP at the health care system level (Figure 1) increased by an order of magnitude from a median of 15.9 (IQR 8.4‐25.5) per 1000 patients in FY 2020 to a median of 227.6 (IQR 127.1‐320.7) per 1000 patients in FY 2023. Secure messages at the health care system level increased by 50% from a median of 23.4 (IQR 9.4‐65.5) per 1000 patients in FY 2020 to 35.3 (IQR 11.0‐87.0) per 1000 patients in FY 2023. Video visits peaked in FY 2021 (median 251.5, IQR 157.4‐279.7 per 1000 patients) and secure messages peaked in FY 2022 (median 46.8, IQR 16.5‐127.8 per 1000 patients) across health care systems. Over the entire 4-year study period, the median video visit volume was 183.2 (IQR 66.9‐323.0) per 1000 patients and the median secure message volume was 37.8 (IQR 13.7‐92.93) per 1000 patients.

**Table 1.** Characteristics of Veterans Health Administration (VHA) primary care providers in the fiscal years 2020 to 2023 (N=17,034).

Characteristics	Providers reporting burnout (n=8574)	Providers reporting no burnout (n=8460)	Total (N=17,034)	*P* value^a^
Age group (y), n (%)				<.001
30‐39	1418 (16.5)	1278 (15.1)	2696 (15.8)	
40‐49	2433 (28.3	2298 (27.2)	4731 (27.8)	
50‐59	3426 (40.0)	3314 (39.2)	6740 (39.6)	
≥60	1297 (15.1)	1570 (18.6)	2867 (16.8)	
Sex, n (%)				<.001
Male	2998 (35.0)	3367 (39.8)	6365 (37.4)	
Female	5255 (61.3)	4934 (58.3)	10,189 (59.8)	
Other or unknown	321 (3.7)	159 (1.9)	480 (2.8)	
Race or ethnicity, n (%)				<.001
Hispanic	665 (7.8)	630 (7.4)	1295 (7.6)	
Non-Hispanic Asian	1254 (14.6)	1495 (17.7)	2749 (16.1)	
Non-Hispanic Black	490 (5.7)	554 (6.5)	1044 (6.1)	
Non-Hispanic other	2163 (25.2)	1947 (23.0)	4110 (24.1)	
Non-Hispanic White	3863 (45.1)	3711 (43.9)	7574 (44.5)	
Non-Hispanic unknown	139 (1.6)	123 (1.5)	262 (1.5)	
VHA tenure (y), n (%)				<.001
<2	1569 (18.3)	1908 (22.6)	3477 (20.4)	
2 to <10	4013 (46.8)	3500 (41.4)	7513 (44.1)	
10 to <20	2093 (24.4)	1944 (23.0)	4037 (23.7)	
≥20	899 (10.5)	1108 (13.1)	2007 (11.8)	
Health care system complexity, n (%)				.007
High	6811 (79.4)	6869 (81.2)	13,680 (80.3)	
Medium	909 (10.6)	855 (10.1)	1764 (10.4)	
Low	854 (10.0)	736 (8.7)	1590 (9.3)	

a Pearson chi-square test was used to generate *P* values for differences between categories.

Across the 4-year study period, burned-out PCPs practiced in health care systems with higher video visits per 1000 patients (median 195.1, IQR 81.4‐332.7) than those who did not report burnout (median 170.0, IQR 45.3‐317.5; Wilcoxon rank-sum test: *P*<.001). Burned-out PCPs also practiced in health care systems with higher secure messages per 1000 patients (median 40.2, IQR 15.0‐97.2) than those without burnout (median 36.7, IQR 13.0‐77.0; Wilcoxon rank-sum test: *P*<.001).

### Adjusted Model Results

In our fully adjusted models (Table 2), each additional secure message per 1000 patients in health care systems was related to a 0.1% increase in burnout (OR 1.001, 95% CI 1.000-1.002) among practicing PCPs. The volume of video visits in health care systems was not significantly related to burnout among practicing PCPs in any quartile of visit volumes. Burnout among all PCPs, in the secure message model, was higher among cross-sections in FY 2021 (OR 1.39, 95% CI 1.28-1.51), in FY 2022 (OR 1.53, 95% CI 1.39-1.69), and in FY 2023 (OR 1.40, 95% CI 1.13-1.74), than in FY 2020. PCP and team staffing ratios at the health care system level are described in Table S2 in Multimedia Appendix 1, and fully adjusted model results with these additional covariates are shown in Table S3 in Multimedia Appendix 1. ORs for the associations between PCP burnout and video visit or secure message volumes in health care systems were unchanged (secure messages) or remained nonsignificant (video visits) from those found in the main analytic models.

**Table 2.** Odds ratios (ORs) and 95% CIs of burnout among the Veterans Health Administration (VHA) primary care providers using logistic regression in fiscal years 2020 to 2023 (N=17,034).

Characteristics	Individual burnout (n=17,034 in 138 healthcare systems), OR (95% CI)			
	Video visit model		Secure message model	
Exposures				
Health care system–level video visit per 1000 patients				
First quartile (0‐38.2 visits)	Reference		—^a^	
Second quartile (38.2‐140.1 visits)	1.01 (0.83-1.24)		—	
Third quartile (140.1‐270.0 visits)	0.97 (0.77-1.23)		—	
Fourth quartile (>270.0 visits)	1.02 (0.79-1.32)		—	
Health care system–level secure messages per 1000 patients	—		1.001^b^ (1.000-1.002)	
Controls				
Fiscal year				
2020	Reference		Reference	
2021	1.43^b^ (1.15-1.77)		1.39^c^ (1.28-1.51)	
2022	1.59^c^ (1.28-1.97)		1.53^c^ (1.39-1.69)	
2023	1.43^d^ (1.05-1.95)		1.40^b^ (1.13-1.74)	
PCP^e^ age (y)				
30‐39	Reference		Reference	
40‐49	0.91 (0.80-1.03)		0.90 (0.80-1.02)	
50‐59	0.90 (0.79-1.03)		0.90 (0.78-1.02)	
≥60	0.74^c^ (0.63-0.88)		0.74^c^ (0.62-0.88)	
PCP sex				
Male	Reference		Reference	
Female	1.17^c^ (1.08-1.26)		1.17^c^ (1.09-1.27)	
Other or unknown	1.93^c^ (1.53-2.45)		1.93^c^ (1.52-2.44)	
PCP race or ethnicity				
Hispanic	0.97 (0.81-1.15)		0.95 (0.80-1.13)	
Non-Hispanic Asian	0.78^c^ (0.69-0.89)		0.78^c^ (0.69-0.88)	
Non-Hispanic Black	0.84^d^ (0.70-1.00)		0.82^d^ (0.68-0.97)	
Non-Hispanic other	1.05 (0.80-1.37)		1.04 (0.79-1.36)	
Non-Hispanic White	Reference		Reference	
Non-Hispanic unknown	0.96 (0.77-1.20)		0.95 (0.76-1.19)	
PCP VHA tenure (y)				
<2	Reference		Reference	
2-<10	1.42^c^ (1.29-1.58)		1.42^c^ (1.28-1.58)	
10-<20	1.41^c^ (1.23-1.62)		1.41^c^ (1.23-1.62)	
≥20	1.11 (0.96-1.28)		1.13 (0.98-1.30)	
Health care system complexity				
High	Reference		Reference	
Medium	1.04 (0.92-1.18)		1.04 (0.93-1.17)	
Low	1.14 (0.94-1.37)		1.16 (0.96-1.39)	

a Not available.

b *P*<.01.

c *P*<.001.

d *P*<.05.

e PCP: primary care provider.

Subgroup analyses by sex are presented in Tables S4 and S5 in Multimedia Appendix 1. Male PCPs with the second and third quartiles of video visit volume were significantly less likely to be burned out than female PCPs in those categories (second quartile: *P*=.02; third quartile: *P*=.007). The probability of PCP burnout by secure message volume in a health care system did not differ by PCP sex but was still significant within each sex.

In our predicted marginal estimates, burnout among PCPs ranged from 48.8% (95% CI 47.0% to 50.5%) without any secure messages per 1000 patients in a health care system to 50.8% (95% CI 49.3% to 52.4%) for 92 secure messages (75th percentile of message volumes) to 53% (95% CI 50.6% to 55.5%) for 191 secure messages (90th percentile of message volumes). PCP burnout was significantly different between each pairwise comparison of the 0th, 75th, and 90th percentiles of secure message volumes in health care systems (*P*=.004 for each comparison). On average, PCP burnout increased by 1% point for each additional increase of 43.7 (95% CI 14.0-73.4) secure messages per 1000 patients in a health care system. This increase in secure messages represents the difference between the median and the 74th percentile of message volumes. Burnout varied less widely by video visit volume in a health care system, with 49.4% (95% CI 47.1% to 51.7%) of PCPs being burned out without any video visits per 1000 patients in a health care system to 50.7% (95% CI 48.9% to 52.6%) of PCPs experiencing burnout with 323 video visits (75th percentile of visit volumes) to 51.6% (95% CI 48.4% to 54.9%) of PCPs experiencing burnout with 527 video visits (90th percentile of visit volumes). Burnout did not significantly differ between these percentiles of video visit volumes in health care systems.

Burnout also varied by demographic characteristics. In this secure message model, burnout was lower among providers aged above 60 years (OR 0.74, 95% CI 0.62-0.88) compared to those aged 30 years. Female (OR 1.17, 95% CI 1.09-1.27) and other sex (OR 1.93, 95% CI 1.52-2.44) PCPs were more burned out than male PCPs. Non-Hispanic Black (OR 0.82, 95% CI 0.68-0.97) and non-Hispanic Asian (OR 0.78, 95% CI 0.69-0.88) PCPs were less likely to be burned out when compared to non-Hispanic White PCPs. Conversely, PCPs with 2 to 10 years of VHA tenure (OR 1.42, 95% CI 1.28-1.58) and 10 to 20 years of VHA tenure (OR 1.41, 95% CI 1.23-1.61) were more likely to be burned out than those with less than 2 years of VHA experience.

## Discussion

### Principal Findings

Video visit and secure message use increased dramatically in VHA health care systems during and after the COVID-19 pandemic, but PCP burnout also increased during this period [6,32]. Our adjusted models show that only high secure message volumes in health care systems were significantly related to high levels of burnout among cross-sections of PCPs practicing there. Our model predicted that the overall burnout rate for PCPs would be associated, on average, with an increase of 1% point for each additional 43.7 secure messages per 1000 patients at the health care system level, which is equivalent to moving from the median to the 74th percentile of secure message volumes. Burnout in our sample also had higher odds among female (vs male), older (vs younger), non-Hispanic White (vs other races), and more tenured (vs less tenured) providers.

To our knowledge, this is the first and largest analysis of the association between PCP burnout and telehealth use. Further research on these relationships is necessary, as our dataset was limited by mixed individual- and health care system–level variables, and our results may be incomplete due to our lack of individual-level telehealth data. Yet, we believe that our analysis is a good first step at understanding how PCP burnout will be affected by future changes in telehealth care.

The increased use of video visits during the pandemic was generally well-received by PCPs [33-35], although there were concerns that this modality could be technically challenging for patients and could reduce the quality of care delivered [36,37]. Video visits or “synchronous telemedicine” in primary care may have been accepted by PCPs because, as noted by a recent scoping review by Lindenfeld et al [38], the modality offers a sufficient substitute to an in-person visit while reducing travel and wait times and increasing access to patients’ family and friends. However, this review noted that video visits cannot sufficiently replace physical examination and may present challenges to patients who have less access or experience with technology, more chronic conditions, and difficulties with verbal or written communication or the English language. According to the job demands-resources model, burnout is exacerbated by increased job demands (eg, high workload) but can be mitigated by increased resources (eg, work control) [21,22]. Video visits may have served as a resource that helped PCPs manage the increased demands they encountered during the COVID-19 pandemic.

On the other hand, secure messages have long been considered an additional job demand in primary care, or a part of “the work no one sees,” as defined by Dyrbye et al [20] in their seminal analysis. VHA patients use secure messaging for a wide variety of information- and communication-based tasks [39,40], and this broad use of the modality may mean that its use increases over time. Some recent estimates by Arndt et al [41,42] found that PCPs in the University of Wisconsin-Madison health care system spent an hour or more a day on secure messages or their “inbox,” with that time spent increasing over the course of the pandemic [42]. Evidence on the association between secure message use and subsequent in-person care is mixed. A panel study conducted in a large private-sector health care system found that patients who sent more messages had over 5% more in-person visits [43]. However, a matched cohort study conducted in the VHA has shown the opposite—patients who were using secure messages had over 15% fewer face-to-face primary care visits [44].

Therefore, it is unclear whether secure message use is a complement or substitute to in-person visits, but that distinction may not matter in the context of burnout. The VHA mandates that 80% of PCP working hours are “bookable” [45] or available for patient encounters. However, only in-person, video, and phone visits, and not secure messages, count as bookable hours. Some private-sector health care systems, like the Cleveland Clinic [46] and UCSF Health [47], do allow PCPs to bill patients or their insurance for responding to secure messages in certain cases. If these responses constitute “medical advice” with regard to their complexity or time-to-completion, PCPs are allowed to bill their responses as a short medical visit. Charging for these messages could also reduce their use and encourage in-person visits. In the VHA, the lack of bookable hour credit for secure messages, and their unclear relationship with future visits, could mean that secure messages, unlike video visits, are related to increased job demands (eg, workload) and reduced job resources (eg, work control) [21,22], and therefore worse PCP burnout.

Female PCPs were more burned out than male PCPs overall and within the second and third quartiles of video visit volume. No sex differences in burnout were found in secure message volume. The disparity in burnout between female and male physicians is well established [7] and may reflect differences in nonwork responsibilities [48] or discrimination and a lack of civility in the workplace [28]. However, there is also evidence that female physicians spend more time with patients during visits and deal with more problems per visit than male physicians [49]. Female physicians also deliver more guideline-based care and use more empathy-based language with patients [50]. In primary care, these behaviors may result in different patient expectations for visits with female PCPs, which could put additional pressure on female PCPs to deliver more time-intensive or emotionally intensive care, leading to burnout. As video visits may be substitutes for in-person visits, even increases in seeing patients by video could be associated with more female PCP burnout.

### Limitations

This study had many strengths, including its analysis of a large, national sample of PCPs from the largest integrated health care system in the United States using repeated annual data from a national health care database and an annual survey with high response rates and stable year-to-year items. However, there were several limitations in our analyses, including the use of cross-sectional rather than longitudinal data. First, the VHA AES contains no interyear identifiers, making longitudinal analyses impossible, but response rates are similar across years, and AES respondents are demographically similar to all VHA employees [51], implying that repeated cross-sectional data are a reasonable substitute for longitudinal data. Our analyses also look at associations between burnout and video visit and secure message volume by year, so intrayear relationships between these variables may be equally well captured by cross-sectional data, even if the respondents change. Second, our data were mixed, with burnout data analyzed at the individual level and video visit and secure message data analyzed at the health care system level. Analyzing only individual-level data was not possible due to the anonymity of the AES data. Some health care systems may have independently promoted or discouraged telehealth use. Therefore, our results show the relationships between individual-level burnout and health care system–level telehealth use and may under- or overstate these relationships due to variations in individual PCP exposure to telehealth. Exploring the associations between PCP-level burnout and video visit and secure message volumes may be appropriate for a future study that collects individual PCP data. Third, these analyses are primarily a result of between-facility associations such that facilities with greater secure message volumes are associated with greater burnout. As a result, there may be additional confounding factors that are not accounted for in our analyses that explain the volume-burnout associations. These additional factors could include variables like individual-level patient complexity, workloads, or administrative burden. However, health care system complexity and a normalized PCP panel size (video visits or secure messages per 1000 patients) may approximate patient complexity and overall or administrative workload for the average PCP.

### Future Research

Improving PCP burnout related to secure messages could involve a variety of solutions, including message inbox automation and rule-based triaging [52], giving PCPs protected time to manage their messages [53], or some other workplace redesign [54]. First, rule-based triaging may simply reduce unnecessary or duplicate messages. Atrius Health, a Massachusetts-based private-sector health care system, was able to reduce daily secure messages to PCPs by 25% through eliminating or redirecting 30% to 98% of messages in the following categories: scanned documents; notes from other physicians; emergency or hospital admission, discharge, or transfer messages; prescription renewals; laboratory results; and patient requests for medical advice [52]. Second, charging patients for message use or allowing PCPs to bill for their time may create an incentive to cut down on messaging or replace messages with in-person visits. As noted earlier, Cleveland Clinic [46] and UCSF Health [47] have started to allow health care providers to bill patients or their insurance for responding to secure messages. However, the effects of workplace redesign and billing for secure messages on reducing PCP burnout have not yet been evaluated. Future research is necessary to determine if these interventions are effective at reducing burnout in practice. Finally, some other workplace redesign may alleviate burnout related to secure messaging. There is some evidence that PCPs who participate in quality improvement interventions to change their workplace are less likely to be burned out [55,56]. Implementing secure message training and support for PCPs could also help improve message use and reduce burnout. It is possible that some changes to secure messaging not described here (eg, the use of artificial intelligence for message summarization) are most effective at reducing PCP burnout or that these changes vary by clinic. In such cases, more flexible changes to the workplace may be more effective in reducing PCP burnout [32], but this flexibility would have to be weighed against the benefits of uniform secure messages policies. Again, the effect of these flexible workplace changes on PCP burnout needs to be evaluated in future research.

### Conclusions

The use of telehealth in VHA primary care increased dramatically during the COVID-19 pandemic, but PCP burnout also increased during this period. However, only the increase in secure message volumes in health care systems, and not video visits, was associated with this rise in burnout among cross-sections of PCPs practicing in these systems. It is possible that video visits acted as substitutes for in-person visits, but that secure messages simply added to the PCP workload. As telehealth use continues to rise in primary care, more research is necessary to ensure that these technologies are not associated with increased PCP burnout.

## Supplementary material

10.2196/68858 Multimedia Appendix 1 Methodological details and additional analyses.

## Abbreviations

AES: All Employee Survey
CDW: Corporate Data Warehouse
FY: fiscal year
odds ratio: OR
PCP: primary care provider
VHA: Veterans Health Administration
