# Supplementary material for: Secure Messages, Video Visits, and Burnout Among Primary Care Providers in the Veterans Health Administration: National Survey Study
Source: J Med Internet Res. 2025 Sep 5;27:e68858. doi: 10.2196/68858 (PMC12413187; doi:10.2196/68858)
Supplement: Multimedia Appendix 1 [file jmir-v27-e68858-s001.docx]

**Appendices.**

**Appendix Table 1. Clinic codes and Current Procedural Terminology modifiers for primary care video and secure message visits**

| **Clinic Code or Current Procedural Terminology (CPT) Modifier** | **Visit Definition** | **Video** | **Secure Message** |
| --- | --- | --- | --- |
| 179 | Real Time Clinical Video Telehealth to Home- Provider Site | X |  |
| 648 | Real Time Clinical Video Telehealth with Non-VA Medical Center Location- Provider Site | X |  |
| 679 | National Center Real Time Clinical Video Telehealth to Home- Provider Site | X |  |
| 95 | Synchronous telemedicine service rendered via a real-time interactive audio and video telecommunications system | X |  |
| 189 | Store & Forward Telehealth from Home- Provider Site |  | X |
| 646 | National Center Store & Forward Telehealth- Patient Site |  | X |
| 647 | National Center Store & Forward Telehealth- Provider Site |  | X |
| 694 | Store & Forward Telehealth- Patient Site |  | X |
| 695 | Store & Forward Telehealth- Provider Site (Same Division/Station) |  | X |
| 696 | Store & Forward Telehealth- Provider Site (Not Same Station) |  | X |
| 698 | Store & Forward Telehealth from Non-VA Medical Center Location- Provider Site |  | X |
| 718 | Diabetic Retinal Screening |  | X |
| 719 | My HealtheVet Secure Messaging |  | X |

**Appendix Table 2. Characteristics of VHA primary care providers including staffing covariates, 2020-2023 (N=17,034)**

| **Characteristic** | **Providers reporting burnout (n=8,574)** | **Providers reporting no burnout (n=8,460)** | **Total (N=17,034)** | **p-value** |
| --- | --- | --- | --- | --- |
| *Age group, n(%)* |  |  |  | <.001 |
| 30-39 | 1,418 (17) | 1,278 (15) | 2,696 (16) |  |
| 40-49 | 2,433 (28) | 2,298 (27) | 4,731 (28) |  |
| 50-59 | 3,426 (40) | 3,314 (39) | 6,740 (40) |  |
| ≥60 | 1,297 (15) | 1,570 (19) | 2,867 (17) |  |
| *Gender, n(%)* |  |  |  | <.001 |
| Male | 2,998 (35) | 3,367 (40) | 6,365 (37) |  |
| Female | 5,255 (61) | 4,934 (58) | 10,189 (60) |  |
| Other/Unknown | 321 (4) | 159 (2) | 480 (3) |  |
| *Race/Ethnicity, n(%)* |  |  |  | <.001 |
| Non-Hispanic White | 3,863 (45) | 3,711 (44) | 7,574 (44) |  |
| Non-Hispanic Black | 490 (6) | 554 (7) | 1,044 (6) |  |
| Non-Hispanic Asian | 1,254 (15) | 1,495 (18 | 2,749 (16) |  |
| Non-Hispanic Other | 2,163 (25) | 1,947 (23) | 4,110 (24) |  |
| Non-Hispanic Unknown | 139 (2) | 123 (1) | 262 (2) |  |
| Hispanic | 665 (8) | 630 (7) | 1,295 (8) |  |
| *VHA Tenure, n(%)* |  |  |  | <.001 |
| <2 years | 1,569 (18) | 1,908 (23) | 3,477 (20) |  |
| 2-<10 years | 4,013 (47) | 3,500 (41) | 7,513 (44) |  |
| 10-<20 years | 2,093 (24) | 1,944 (23) | 4,037 (24) |  |
| ≥20 years | 899 (10) | 1,108 (13) | 2,007 (12) |  |
| *Facility Complexity, n(%)* |  |  |  |  |
| High | 6,811 (79) | 6,869 (81) | 13,680 (80) | 0.007 |
| Medium | 909 (11) | 855 (10) | 1,764 (10) |  |
| Low | 854 (10) | 736 (9) | 1,590 (9) |  |
| *Healthcare system-level percent of teams with staffing ratio >= 3* |  |  |  |  |
| <50% (Very understaffed) | 4,773 (56) | 4,909 (58) | 9,682 (57) | .001 |
| 50-100% (Better staffed) | 3,801 (44) | 3,551 (42) | 7,352 (43) |  |
| *Healthcare system-level provider staffing* |  |  |  |  |
| <1.2 (Not fully staffed) | 1,759 (21) | 1,411 (17) | 3,170 (19) | <.001 |
| >=1.2 (Fully staffed) | 6,815 (79) | 7,049 (83) | 13,864 (81) |  |

**Abbreviation: VHA = Veterans Health Administration**

**Appendix Table 3. Odds ratios and 95% CIs of burnout among VHA primary care providers including staffing covariates using logistic regression, 2020-2023**

|  | **Individual Burnout** | | | |
| --- | --- | --- | --- | --- |
|  | **n=17,034 in 138 healthcare systems** | | **n=17,034 in 138 healthcare systems** | |
| **Characteristic** | **OR** | **95% CI** | **OR** | **95% CI** |
| ***Exposures*** |  |  |  |  |
| *Healthcare system-level video visits per 1,000 patients* |  |  | -- |  |
| 1^st^ quartile (0-38.2 visits) | Ref |  |  |  |
| 2^nd^ quartile (38.2-140.1 visits) | 1.05 | 0.86, 1.28 |  |  |
| 3^rd^ quartile (140.1-270.0 visits) | 0.99 | 0.79, 1.24 |  |  |
| 4^th^ quartile (>270.0 visits) | 1.06 | 0.83, 1.36 |  |  |
| *Healthcare system-level secure messages per 1,000 patients* | -- |  | 1.001* | 1.000, 1.002 |
| ***Controls*** |  |  |  |  |
| *Fiscal Year* |  |  |  |  |
| 2020 | Ref |  | Ref |  |
| 2021 | 1.40** | 1.13, 1.73 | 1.40*** | 1.29, 1.52 |
| 2022 | 1.56*** | 1.27, 1.93 | 1.56*** | 1.42, 1.72 |
| 2023 | 1.40* | 1.03, 1.89 | 1.41** | 1.14, 1.74 |
| *Provider Age* |  |  |  |  |
| 30-39 | Ref |  | Ref |  |
| 40-49 | 0.90 | 0.79, 1.02 | 0.90 | 0.79, 1.02 |
| 50-59 | 0.89 | 0.78, 1.02 | 0.89 | 0.78, 1.02 |
| ≥60 | 0.74*** | 0.62, 0.88 | 0.73*** | 0.62, 0.87 |
| *Provider Gender* |  |  |  |  |
| Male | Ref |  | Ref |  |
| Female | 1.17*** | 1.08, 1.26 | 1.17*** | 1.08, 1.27 |
| Other/Unknown | 1.93*** | 1.53, 2.45 | 1.93*** | 1.52, 2.44 |
| *Provider Race/Ethnicity* |  |  |  |  |
| Non-Hispanic White | Ref |  | Ref |  |
| Non-Hispanic Black | 0.81* | 0.69, 0.97 | 0.80** | 0.67, 0.95 |
| Non-Hispanic Asian | 0.78*** | 0.69, 0.88 | 0.78*** | 0.69, 0.88 |
| Non-Hispanic Other | 1.05 | 0.80, 1.37 | 1.04 | 0.80, 1.36 |
| Non-Hispanic Unknown | 0.96 | 0.77, 1.20 | 0.96 | 0.77, 1.20 |
| Hispanic | 0.97 | 0.82, 1.16 | 0.96 | 0.81, 1.14 |
| *Provider VHA Tenure* |  |  |  |  |
| <2 years | Ref |  | Ref |  |
| 2-<10 years | 1.43*** | 1.29, 1.58 | 1.43*** | 1.29, 1.58 |
| 10-<20 years | 1.42*** | 1.24, 1.62 | 1.42*** | 1.24, 1.63 |
| ≥20 years | 1.13 | 0.98, 1.30 | 1.15 | 1.00, 1.32 |
| *Facility Complexity* |  |  |  |  |
| High | Ref |  | Ref |  |
| Medium | 1.09 | 0.96, 1.24 | 1.08 | 0.96, 1.23 |
| Low | 1.16 | 0.97, 1.39 | 1.17 | 0.98, 1.40 |
| *Healthcare system-level percent of teams with staffing ratio >= 3* |  |  |  |  |
| <50% (Very understaffed) | Ref |  | Ref |  |
| 50-100% (Better staffed) | 1.06 | 0.95, 1.19 | 1.06 | 0.94, 1.19 |
| *Healthcare system-level provider staffing* |  |  |  |  |
| <1.2 (Not fully staffed) | Ref |  | Ref |  |
| >=1.2 (Fully staffed) | 0.75*** | 0.66, 0.85 | 0.77*** | 0.68, 0.87 |

**Note: * = p<0.05, ** = p<0.01, *** = p<0.001; Abbreviations: CI = confidence interval; OR = odds ratio; VHA = Veterans Health Administration**

**Appendix Table 4. Odds ratios and 95% CIs of burnout among VHA primary care providers by video visit volume and gender using logistic regression, 2020-2023**

|  | **Individual Burnout** | | | |
| --- | --- | --- | --- | --- |
|  | **n=6365 male PCPs in 138 healthcare systems** | | **n=10,189 female PCPs in 138 healthcare systems** | |
| **Characteristic** | **OR** | **95% CI** | **OR** | **95% CI** |
| ***Exposure*** |  |  |  |  |
| *Healthcare system-level video visits per 1,000 patients* |  |  | -- |  |
| 1^st^ quartile (0-38.2 visits) | Ref |  |  |  |
| 2^nd^ quartile (38.2-140.1 visits) | 0.76 | 0.55, 1.04 | 1.20 | 0.98, 1.47 |
| 3^rd^ quartile (140.1-270.0 visits) | 0.66* | 0.45, 0.95 | 1.21 | 0.96, 1.52 |
| 4^th^ quartile (>270.0 visits) | 0.77 | 0.53, 1.13 | 1.18 | 0.92, 1.51 |
| ***Controls*** |  |  |  |  |
| *Fiscal Year* |  |  |  |  |
| 2020 | Ref |  | Ref |  |
| 2021 | 1.88*** | 1.34, 2.63 | 1.23 | 0.99, 1.52 |
| 2022 | 1.98*** | 1.41, 2.79 | 1.39** | 1.12, 1.72 |
| 2023 | 1.79* | 1.11, 2.88 | 1.28 | 0.87, 1.90 |
| *Provider Age* |  |  |  |  |
| 30-39 | Ref |  | Ref |  |
| 40-49 | 0.77* | 0.62, 0.95 | 0.95 | 0.80, 1.13 |
| 50-59 | 0.85 | 0.67, 1.07 | 0.90 | 0.78, 1.05 |
| ≥60 | 0.75* | 0.58, 0.97 | 0.71** | 0.57, 0.88 |
| *Provider Race/Ethnicity* |  |  |  |  |
| Non-Hispanic White | Ref |  | Ref |  |
| Non-Hispanic Black | 0.80 | 0.58, 1.10 | 0.84 | 0.70, 1.02 |
| Non-Hispanic Asian | 0.75** | 0.63, 0.89 | 0.79** | 0.68, 0.93 |
| Non-Hispanic Other | 1.23 | 0.79, 1.90 | 0.91 | 0.65, 1.28 |
| Non-Hispanic Unknown | 1.01 | 0.69, 1.47 | 0.91 | 0.66, 1.26 |
| Hispanic | 0.95 | 0.75, 1.22 | 0.97 | 0.76, 1.26 |
| *Provider VHA Tenure* |  |  |  |  |
| <2 years | Ref |  | Ref |  |
| 2-<10 years | 1.43*** | 1.23, 1.66 | 1.43*** | 1.25, 1.64 |
| 10-<20 years | 1.46*** | 1.18, 1.80 | 1.42*** | 1.21, 1.66 |
| ≥20 years | 0.98 | 0.78, 1.22 | 1.28 | 1.06, 1.53 |
| *Facility Complexity* |  |  |  |  |
| High | Ref |  | Ref |  |
| Medium | 1.07 | 0.86, 1.33 | 1.03 | 0.90, 1.18 |
| Low | 1.20 | 0.91, 1.59 | 1.12 | 0.92, 1.36 |

**Note: * = p<0.05, ** = p<0.01, *** = p<0.001; Abbreviations: CI = confidence interval; OR = odds ratio; VHA = Veterans Health Administration**

**Appendix Table 5. Odds ratios and 95% CIs of burnout among VHA primary care providers by secure message volume and gender using logistic regression, 2020-2023**

|  | **Individual Burnout** | | | |
| --- | --- | --- | --- | --- |
|  | **n=6365 male PCPs in 138 healthcare systems** | | **n=10,189 female PCPs in 138 healthcare systems** | |
| **Characteristic** | **OR** | **95% CI** | **OR** | **95% CI** |
| ***Exposure*** |  |  |  |  |
| *Healthcare system-level secure messages per 1,000 patients* | 1.001* | 1.001, 1.002 | 1.0008** | 1.0002, 1.001 |
| ***Controls*** |  |  |  |  |
| *Fiscal Year* |  |  |  |  |
| 2020 | Ref |  | Ref |  |
| 2021 | 1.37** | 1.21, 1.56 | 1.40** | 1.26, 1.55 |
| 2022 | 1.44** | 1.25, 1.66 | 1.57** | 1.39, 1.77 |
| 2023 | 1.30 | 0.92, 1.84 | 1.48* | 1.05, 2.08 |
| *Provider Age* |  |  |  |  |
| 30-39 | Ref |  | Ref |  |
| 40-49 | 0.77* | 0.63, 0.95 | 0.95 | 0.80, 1.13 |
| 50-59 | 0.85 | 0.67, 1.07 | 0.90 | 0.77, 1.05 |
| ≥60 | 0.74* | 0.57, 0.96 | 0.71** | 0.57, 0.88 |
| *Provider Race/Ethnicity* |  |  |  |  |
| Non-Hispanic White | Ref |  | Ref |  |
| Non-Hispanic Black | 0.77 | 0.56, 1.07 | 0.82 | 0.68, 0.99 |
| Non-Hispanic Asian | 0.75** | 0.63, 0.89 | 0.79** | 0.67, 0.93 |
| Non-Hispanic Other | 1.21 | 0.78, 1.87 | 0.92 | 0.65, 1.29 |
| Non-Hispanic Unknown | 1.02 | 0.70, 1.49 | 0.91 | 0.66, 1.25 |
| Hispanic | 0.94 | 0.74, 1.19 | 0.95 | 0.74, 1.23 |
| *Provider VHA Tenure* |  |  |  |  |
| <2 years | Ref |  | Ref |  |
| 2-<10 years | 1.42*** | 1.22, 1.65 | 1.43*** | 1.25, 1.64 |
| 10-<20 years | 1.45*** | 1.17, 1.79 | 1.42*** | 1.21, 1.67 |
| ≥20 years | 1.00 | 0.80, 1.25 | 1.29** | 1.07, 1.55 |
| *Facility Complexity* |  |  |  |  |
| High | Ref |  | Ref |  |
| Medium | 1.07 | 0.87, 1.32 | 1.03 | 0.90, 1.18 |
| Low | 1.23 | 0.93, 1.63 | 1.13 | 0.94, 1.38 |

**Note: * = p<0.05, ** = p<0.01, *** = p<0.001; Abbreviations: CI = confidence interval; OR = odds ratio; VHA = Veterans Health Administration**
